# Interstitial Fluid Flows along Perivascular and Adventitial Clearances around Neurovascular Bundles

**DOI:** 10.3390/jfb13040172

**Published:** 2022-10-01

**Authors:** Yiya Kong, Xiaobin Yu, Gang Peng, Fang Wang, Yajun Yin

**Affiliations:** 1 Department of Cardiology, Beijing Hospital, National Center of Gerontology, Institute of Geriatric Medicine, Chinese Academy of Medical Sciences, Beijing 100730, China; 2 Graduate School, Peking Union Medical College, Chinese Academy of Medical Sciences, Beijing 100730, China; 3 Department of Engineering Mechanics, Tsinghua University, Beijing 100084, China

**Keywords:** interstitial fluid (ISF), microflow, perivascular and adventitial clearance, neurovascular bundles, accompanying vein, accompanying artery, kinematic law

## Abstract

This study reports new phenomena of the interstitial fluid (ISF) microflow along perivascular and adventitial clearances (PAC) around neurovascular bundles. The fluorescent tracing was used to observe the ISF flow along the PAC of neurovascular bundles in 8–10 week old BALB/c mice. The new results include: (1) the topologic structure of the PAC around the neurovascular bundles is revealed; (2) the heart-orientated ISF flow along the PAC is observed; (3) the double-belt ISF flow along the venous adventitial clearance of the PAC is recorded; (4) the waterfall-like ISF flow induced by the small branching vessel or torn fascia along the PAC is discovered. Based on the above new phenomena, this paper approached the following objectives: (1) the kinematic laws of the ISF flow along the PAC around neurovascular bundles are set up; (2) the applicability of the hypothesis on the PAC and its subspaces by numerical simulations are examined. The findings of this paper not only enriched the image of the ISF flow through the body but also explained the kernel structure of the ISF flow (i.e., the PAC). It helps to lay the foundation for the kinematics and dynamics of the ISF flow along the PAC around neurovascular bundles.

## 1. Introduction

The interstitial fluid (ISF) is a vital body fluid thatconnects with the blood, lymphatic fluid, and intracellular fluid. In the traditional physiological view, the majority of ISF is bound within the hydrogel-like extracellular matrix that cannot flow freely [1]. This study reports new phenomena of the interstitial fluid (ISF) microflow along perivascular and adventitial clearances (PAC) around neurovascular bundles. 

Recently, the dynamic flow of the ISF along the perivascular space (PVS) has caused ongingconcern. The PVS, also known as the “Virchow-Robin space”, is the space that surrounds the parenchymal vessels, with the glial membrane as the outer boundary and the vascular wall as the inner boundary. The PVS, together with the entry of penetrating arteries and the exit of draining veins from the cerebral cortex, is an ISF or cerebrospinal fluid (CSF)-filled, pial-lined structure [2,3]. Iliff et al. named the efficient convective exchange clearance system of CSF and ISF along the PVS as the “glymphatic system”, which relies on astrocytes via aquaporin-4 in the central nervous system [4,5]. Its function has been demonstrated successively in human and animal models in association with various neurological disorders [6,7]. The diameter of cerebral PVS in mice was measured to be approximately 40 μm, and its integrated cross-section area was approximately 1.4 times that of the adjacent vessels found by two-photon microscopy [8,9]. The force to drive the cerebral ISF flow in the glymphatic system is considered to be significantly correlated with arterial pulsation but not with respiratory movement [4,10]. 

Carare et al. regarded the basement membranes of cerebral capillaries and arteries as the counterpart of cerebral lymphatic pathways. The drainage of the ISF occurs along the intramural periarterial drainage (IPAD). However, no empirical evidence of tracers coming into and out of the parenchyma was found on cortical veins [11,12,13]. The vasodilation movements produced by contraction and diastole of cerebrovascular smooth muscle cells are the main drivers of IPAD [14]. 

Until the recent studies of cerebral ISF flow, the available experimental evidence does not provide an adequate description of how the venous walls and the surrounding space drain the ISF, compared to the arteries. In any case, the functional role of venous drainage of the ISF appears to be minor [15]. Apart from the brain, Li et al. demonstrated that long-distance ISF flow exists in the arterial and venous adventitia and their surrounding fibrous connective tissues throughout the whole body, and possibly also in the epineurium and skin [16,17].

The aforementioned studies all focused on isolated blood vessels, which were not able to explain the experimental phenomenon (i.e., the ISF flow within the perivascular) in this study. Meanwhile, it should be emphasized that the majority of veins (excluding special superficial venous) are accompanied by an artery and a nerve (or nerves) to form neurovascular bundles [18]. The specificity and integrality of neurovascular bundles’ structure should be considered to provide a complete and less contradictory basis for ISF flow dynamics. Consequently, accompanying neurovascular bundles are observed as a holistic object in this study to locate the ISF flow space, and the frame with which the diverse ISF flow patterns can be interpreted consistently.

The paper is organized as follows. Section 4 introduces our experimental materials and methods. Section 2 shows the experimental results, including (a) the structure of the PAC, (b) the diverse ISF microflow patterns inside the PAC, and (c) the novel phenomenon of the waterfall-like ISF flow. Section 3 refined the kinematic laws of the ISF flow along the PAC around neurovascular bundles, based on the three kinematic laws of ISF flow over isolated arteries and veins [19].

These findings have not only enriched the image of the ISF flow through the body (except in the brain), but have also explained the kernel structure of the ISF flow (i.e., PAC). Only by clarifying the kernel structure of the ISF flow can we further study the pathways and properties of the ISF flow and elucidate the kinematic mechanism. It helps to develop the kinematics and dynamics of the ISF microflow along the PAC around neurovascular bundles.

## 2. Results

### 2.1. Fluorescence-Stained and Histological Analysis of the PAC Structure around Neurovascular Bundles

**Figure 1:**
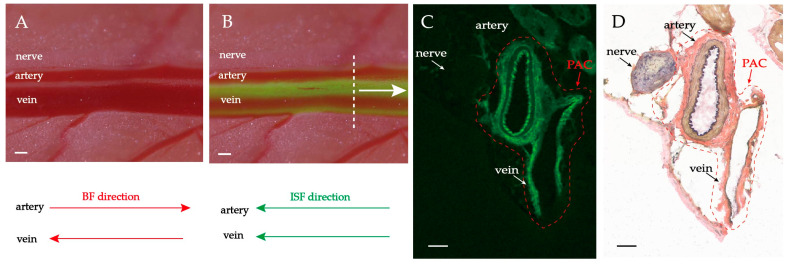
ISF flow along PAC around neurovascular bundles. (**A**) Brightfield observation before fluorescing. (**B**) Brightfield observation with fluorescent lamp opening after fluorescing. The heart direction is located left. Scale bar: 200 μm. (**C**,**D**) Frozen fluorescent sections and EVG staining show the structure of PAC. Magnification 20×. The arrows indicate nerve, artery, vein, and PAC. Scale bar: 50 μm.

5.0 μL fluorescent tracer was supplied. The observed site is 1.5–2.0 cm away from the supply point (Figure 1A). The flow pathways have been fluorescently stained along the direction of the ISF flow. The ISF flows towards the heart in the same direction as venous blood flow, and opposite to arterial blood flow (Figure 1B). The fluorescent-stained neurovascular bundles were subjected to rapidly frozen sectioning and Verhoeff’s Van Gieson (EVG) staining.

**Figure 2:**
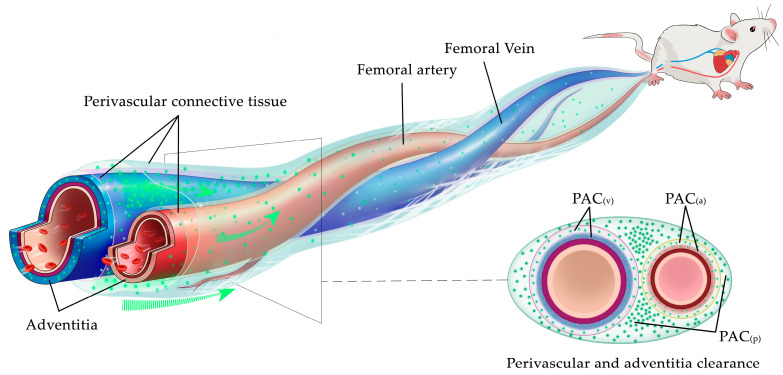
The observed space for ISF flow along PAC around neurovascular bundles. Perivascular and adventitial clearance (PAC) can be divided into arterial adventitial clearance (PAC(a)), venous adventitial clearance (PAC(v)) and perivascular connective tissue flow space (PAC(p)).

The observed ISF flows mainly occur in three areas, namely: the venous adventitia, the arterial adventitia, and the connective tissue surrounding the accompanying artery and vein. The entire flow space is termed perivascular and adventitial clearance (abbreviated as PAC). Furthermore, the PAC can be more finely divided into three subspaces, namely arterial adventitial clearance (PAC(a)), venous adventitial clearance (PAC(v)), and perivascular connective tissue flow space (PAC(p)) (Figure 2).

The histological results show that PAC(a), PAC(v), and PAC(p) were fluorescence-stained (Figure 1C). It is worth noting that soluble fluorescent tracers can penetrate and migrate into the medial and intima layers of the artery and vein. Therefore, the presence of fluorescent signals in the media smooth muscle and the intimal layer does not mean that they are all the natural flow pathways of the ISF. Corresponding to the EVG staining (Figure 1D), there is abundant loose connective tissue around the artery, the vein, and the nerve. The vascular adventitia itself is also loose connective tissue, with no clear boundary between them and the surrounding loose connective tissue. The EVG results show that the diameter of the PAC(a) is approximately 200 μm (n ≥ 5), PAC(v) is approximately 400 μm (n ≥ 5), the nerve is approximately 200 μm (n ≥ 5), and the PAC is approximately 600–750 μm (n ≥ 5).

The ISF flow pattern and the topological structure of the PAC shown in Figure 1 are universalities. To properly depict the ISF flow phenomena along the PAC in the following experiments, a general diagram of the PAC is abstracted from Figure 1 and displayed in Figure 2. The fluorescent spots in Figure 2 mark the ISF flow spaces. From Figure 2, we can predict that once the relative positions between the outer connective tissue membrane and adventitia change, both the ISF flow spaces and the flow patterns will change. This prediction is confirmed in the following experiment.

### 2.2. Heart-Orientated ISF Flow along PAC(p)

**Figure 3:**
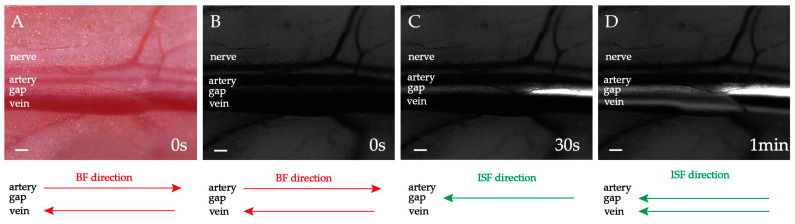
ISF flow along PAC(p). (**A**) Brightfield observation before fluorescing. (**B**) Fluorescence observation before fluorescing. (**C**,**D**) Fluorescence observation after fluorescing. The heart is located left. The ISF flow is pointed to the heart. Scale bar: 200 μm.

1.0 μL fluorescent tracer was supplied. The femoral artery and vein observed are 1.5–2.0 cm away from the supply point (Figure 3A,B). The fluorescent ISF flows along the connective tissue gap (i.e., PAC(p)) between the accompanying femoral artery and vein after a few seconds (Figure 3C and Supplementary Materials Video S1). Meanwhile, there is no fluorescent signal over PAC(a) or PAC(v) (Figure 3C). Then the fluorescent signal began to appear on the vein (Figure 3D). At the same time, in the gap between the accompanying artery and vein, the fluorescence is brighter, and the width of the fluorescence belt is wider (Video S1). The direction of the ISF flow is right-to-left and pointed to the heart. The flow direction towards the heart is always the same as the venous blood flow. In short, the PAC(p) is one of the ISF’s directional flow channels. Because the ISF mainly flows inside the PAC(p) or the gap between the accompanying vein and artery, we may term the phenomenon “gap-flow”. The gap width is about 300 μm, so the gap-flow is a continuous microflow.

### 2.3. Double-Belt ISF Flow along PAC(v) around Neurovascular Bundles

**Figure 4:**
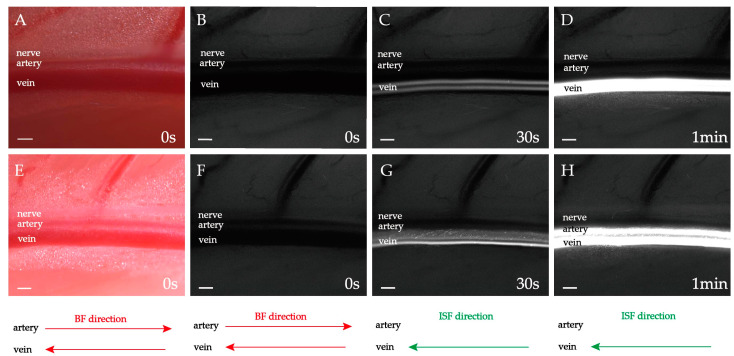
Double-belt ISF flow along PAC(v). (**A**–**D**) Equal-width double-belt ISF flow; (**E**–**H**) Unequal-width double-belt ISF flow. (**A**,**E**) Brightfield observation before fluorescing. (**B**,**F**) Fluorescence observation before fluorescing. (**C**,**D**,**G**,**H**) Fluorescence observation after fluorescing. The heart is located left. The ISF flow is pointed to the heart. Scale bar: 200 μm.

1.0 μL fluorescent tracer was supplied. The femoral artery and vein observed are 1.5–2.0 cm away from the supply point (Figure 4A,B). After a few seconds, two parallel “fluorescence flow belts (FFB)” with equal width appeared simultaneously along the PAC(v) (Figure 4C). Each FFB is approximately 1/3 of the venous diameter (Figure 4C). As time went on, both FFBs widened, and the brightness increased. The two FFBs gradually fused into a single one after about 1 minute (Figure 4D and Video S2). The direction of the ISF flow is right-to-left, pointed to the heart.

The flow phenomenon in Figure 4 is termed “double-belt flow”. Aside from the equal-width double-belt flows, unequal-width double-belt flows were also similarly observed.

Two parallel FFBs with unequal widths appear simultaneously along the PAC(v) a few seconds after the fluorescent tracer was supplied (Figure 4F). The two FFBs are approximately 1/4 and 1/2 width of the venous diameter, respectively. Both FFBs widen, and their brightness increases with time. After 1 minute, the two FBs gradually fused into one (Figure 4G and Video S3).

Both the equal and unequal double-belt flows along the PAC(v) around neurovascular bundles are rapid directional ISF flows.

How do we explain the double-belt ISF flow pattern? The relative locations of the adventitia and the multi layers of perivascular connective tissue may provide the answers. If the multi layers of connective tissue have adhered to the venue adventitia—such adhesion always occurs in neurovascular bundles—then an adhesion belt (i.e., the black no-flow belt) in Figure 4C may be formed. This adhesion belt will separate the flow space along the venue adventitia into two parts, where the two streams of ISF (i.e., the white flow belt) are observed.

### 2.4. Waterfall-like ISF Flow Induced by the Small Branching Vessel or Torn Fascia along PAC

The above experiments display the smooth ISF flows along the PAC. In our experiments, we also captured the various unsmooth ISF flows along the PAC. Next, the unsmooth flows caused by obstacles in the ISF flow pathway were reported.

**Figure 5:**
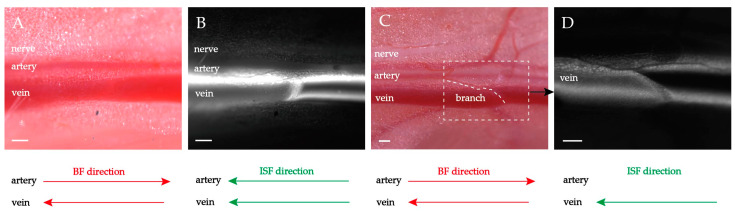
The small branching vessel or torn fascia induced waterfall-like ISF flow along PAC. (**A**,**B**) Torn fascia across the vein induced waterfall-like ISF flow. (**C**,**D**) The small vessel across the vein induced a waterfall-like ISF flow. (**A**,**C**) Brightfield observation before fluorescing. (**B**,**D**) Fluorescence observation after fluorescing. The heart is located in the left direction. The ISF flow is pointed to the heart. Scale bar: 200 μm.

1.0 μL fluorescent tracer was supplied. Using ophthalmic forceps, we gently tear the fascia formed by connected tissue covering the outermost layer of the femoral vein. A fascia layer is torn across the middle of the vein, 1.5–2.0 cm away from the supplying point (Figure 5A). Although the outer membrane of the multi-layered fascia was torn, the left layers can still ensure that the PAC structure is integral. When the fluorescent ISF flows, a fissure fascia is observed across the middle of the vein, and a waterfall-like ISF flow is induced along the PAC (Figure 5B and Video S4).

The same effect will also be created when a small nourishing vessel crosses over the main vein (Figure 5C). The waterfall-like ISF flow along the PAC is induced by the small branching vessel crossing the central part of the vein (Figure 5D). The ISF can be seen to overturn the small branching vessel to create an angled waterfall-like flow across the main vein (Video S5). 

The Supplementary Video shows that the partial ISF is modulated by the small vessel and flows along the branching. It can be deduced that the ISF flow is massive enough to form a local waterfall-like flow by any obstructive barrier in the PAC.

## 3. Discussion

### 3.1. The PAC Is the Kernel Structure for ISF Flow

Unlike the classical intraluminal flow (e.g., blood, lymphatic fluid), which is well known in traditional physiology, the ISF flow is a particular non-luminal flow. What is the flow space of this kind of non-luminal fluid is an essential and complicated key issue and the cornerstone of in-depth research. Only by clarifying the kernel structure of the ISF flow can we further study the pathways and properties of the ISF flow and elucidate the kinetic mechanism. 

As a flow space for the ISF, the PAC in the body and the PVS in the brain are comparable. In the glymphatic system, the cerebral ISF flow space is believed to be the PVS. However, there are still many controversies. Prior to in vivo two-photon imaging, the existence of the PVS was doubted. There are significant morphological differences in the PVS when observed in vivo and dead states. The PVS may disappear due to dehydration, and the tracer was deposited into the adjacent collagen fibers when the animal died or during the tissue fixation process [9,20]. The research evidence on the drainage of the cerebral ISF along the IPAD pathway was controversial: (1) The results of the tissue sections showed that the tracer was deposited into the smooth muscle and basement membrane layers, but whether the region of tracer deposition marked the real ISF flow was inexplicable [21]. (2) Whether the pressure within the IPAD was able to drive a steady flow, and if the water resistance within the IPAD is so big that it can accommodate a large cerebral ISF, are all unclear [22]. These controversies are sufficient to show that revealing the kernel structure of the ISF flow is a very complex matter. However, it must be pointed out that when comparing the PAC and PVS, there is a need to note that the ISF flow environment in the lower limbs is quite different from that in the brain. For instance, (1) The brain has close to 80% of high water content [23]. The brain’s extracellular fluids consist of ISF, blood plasma, and cerebrospinal fluid (CSF). CSF is also involved in the glymphatic system. (2) The blood–brain barrier (BBB) is a particular anatomic and physiologic barrier separating the circulating blood from the extracellular fluid in the central nervous system [24]. We have tried to elucidate the kernel structure of the ISF flow in the lower limbs, which is the most crucial purpose of this article. The cerebral ISF flow has its characteristics, but the ISF flow also occurs in the whole body. We chose to focus on blood vessels in the lower limbs because femoral arteries and veins are easily exposed, which facilitates real-time observation in vivo. (2) Unlike injection tracer into the cisterna magna, we could achieve a relatively quantitative and direct supply of fluorescent tracer to trace pathways in the lower limbs.

Our study revealed the new phenomena of ISF flow along vessel walls and in the surrounding space. The vascular adventitia is mainly composed of loose connective tissue containing extracellular matrix (ECM), fibroblasts and small perivascular nerves, in which loosely arranged, spiral or longitudinally-distributed collagen, elastic and reticular fibers form a stress-bearing viscoelastic skeletal structure filled with large amounts of amorphous material (e.g., hyaluronic acid) [25]. The perivascular space of both arteries and veins is also surrounded by abundant loose connective tissue. It is difficult to draw a clear boundary between the adventitia of blood vessels and their surrounding connective tissue (Figure 1D). The connective tissue becomes looser as it moves outwards, centered on the blood vessels [26,27]. The abundant loose connective tissue along the vascular walls provides a flowable space for a large amount of ISF flow within the PAC (Figure 2).

As mentioned above, the PAC includes three regions (i.e., PAC(a), PAC(v) and PAC(p), see Figure 2). The three subspaces are not three mutually exclusive subsets but are interconnected and complementary. Because of the diversity of biological structures and individual differences, when one subspace in the PAC is looser than another, its flow resistance will be smaller and the ISF will flow relatively faster. Therefore, when a very small amount of fluorescent tracer is supplied, sometimes it seemed that only one of the subspaces was observed to be brightened. However, the real flow should occur in all subspaces at the same time. In addition, for no-blood-vessel-accompanying nerve bundles or only the artery accompanying the vein, the three subspaces are not necessarily present simultaneously. They are combined by a subset of them, so the flow space on an isolated artery (vein) can be formed by the combination of PAC(a) or PAC(a) + PAC(p) (PAC (a ) or PAC(v) + PAC(p)).

Up until now, most flows have been observed along the PAC. There is no clear answer as to whether there is an ISF flow along the neural epithelium. The reason might be that the dynamic images of fluorescent ISF flowing along the neural epithelium in vivo were not captured due to the limitation of the fluorescence stereo microscopes. However, the static images given in the previous study by Li et al. showed that the neural epithelium was fluorescently brightened in some situations. More experimental evidence is still required. Whatever the case, it is certain that the accompanying nerve provides a partial boundary for the ISF flow along the PAC. Therefore, we adopted a holistic perspective, using the neurovascular bundles as the object of study. It is possible to clarify the basic structure of the PAC to understand the diversity of the ISF flow patterns.

### 3.2. Spontaneous ISF Flow Instead of Pressure-Driven Flow or Tracer Diffusion

**Figure 6:**
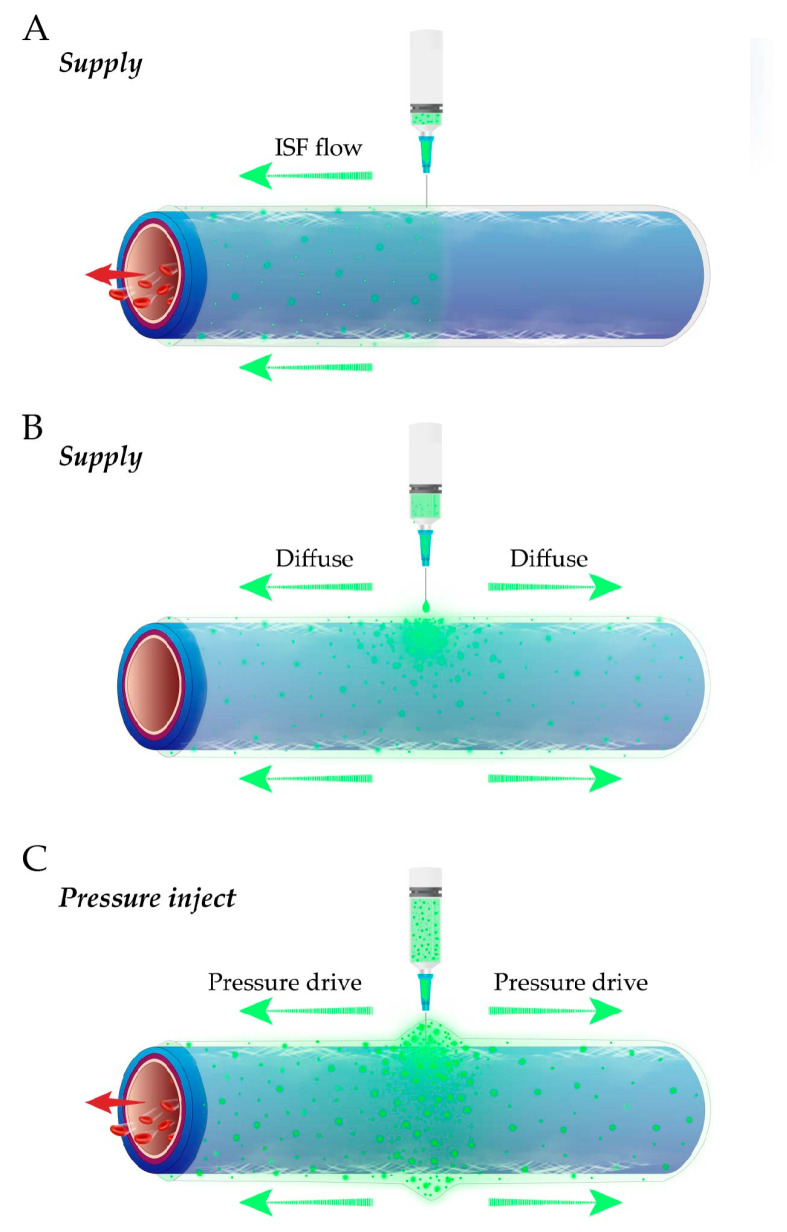
Three different fluorescent tracer approaches. (**A**) Supplying fluorescent tracer in vivo. (**B**) Supplying fluorescent tracers in the dead state. (**C**) Pressure injection in vivo.

The shown ISF flow along the PAC is spontaneous. In other words, the ISF flow is not driven by external pressure such as injection, and it is not diffusion. The reason is as follows. In our all experiments, the fluorescent tracer was supplied with minimal amounts (1.0–5.0 μL), slowly and with no pressure in vivo (Figure 6A). This approach differs from pressure injection, which may cause the fluorescent tracer to flow simultaneously to the heart and far from the heart (Figure 6C). 

Moreover, in the dead state, supplying the fluorescent tracer may produce diffusion in all directions (Figure 6B). Supplementary Experiment-1 was provided to repeat supplying in the dead state. In Supplementary Experiment-1, the 0.5 μL fluorescent tracer was supplied to the vascular adventitia in the middle of the exposed vessels in the dead state of mice, and we found that the fluorescent ISF was diffused (Figure S1).

### 3.3. The Diversity of ISF Flow Patterns within PAC

**Figure 7:**
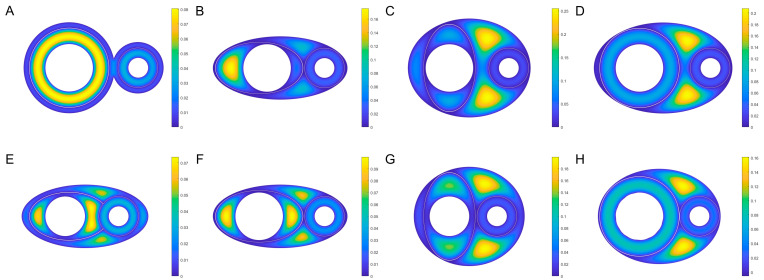
Simulated ISF flowing patterns. Three separated flowing spaces are PAC(a), PAC(v) and PAC(p) respectively. (**A**–**H**) Flowing velocity distribution on different cross-section geometry. On the boundaries, the velocity is zero without permeating. Flow velocity decreases from yellow to blue.

To investigate the effect of the geometry of the arteriovenous and their surrounding fascia on the flow distribution, simplified models are considered. For example, the cross-section shown in Figure 7, where the circle and the ellipse characterize the outer fascia and the arteriovenous membranes, respectively. At the small Reynolds number, the viscous laminar flow in the fully developed and eventually formed a constant flow. As this study focuses on the flow space rather than the dynamic pattern, the model ignores the changes in the longitudinal direction of the vessel due to pulsatile or systaltic motion. The z-related terms in the nonlinear Navi-Stokes equations all degenerate to zero, and the equations degenerate to a two-dimension Poisson’s equation. The flow velocity distribution problem within the cross-section becomes a Poisson equation boundary value problem under Dirichlet boundary conditions, with the governing equation given by

(1) $$\frac{\partial^{2}u_{z}}{\partial x^{2}}+\frac{\partial^{2}u_{z}}{\partial y^{2}}=\frac{1}{\mu }\frac{dp}{dz}.$$

where: (x,y,z) represents the spatial position coordinate; $u_{z}$ is the velocity in the z-direction; μ is the kinematic viscosity; $\frac{dp}{dz}$ is the pressure gradient along the z-axis.

The arterial radius $r_{a}$ is selected as the characteristic length, with the characteristic velocity denoted as $\frac{r_{a}{}^{2}}{\mu }\frac{dp}{dz}$, then the following dimensionless parameters can be introduced

(2) $$\xi =\frac{x}{r_{a}},\;\eta =\frac{y}{r_{a}},\;u=u_{z}\frac{\mu }{r_{a}{}^{2}}\frac{\mathrm{dz}}{\mathrm{dp}}.$$

The governing equation then becomes

(3) $$\frac{\partial^{2}u}{\partial \xi^{2}}+\frac{\partial^{2}u}{\partial \eta^{2}}=1$$

On the outer boundary, the transmembrane permeation rate $u_{x}$ and $u_{y}$ are small compared with the longitudinal velocity. As a solid fibrous structure, the fascia could not flow itself, so the boundary condition can be considered as a Dirichlet boundary, i.e., u=0 on the boundary.

**Figure 8:**
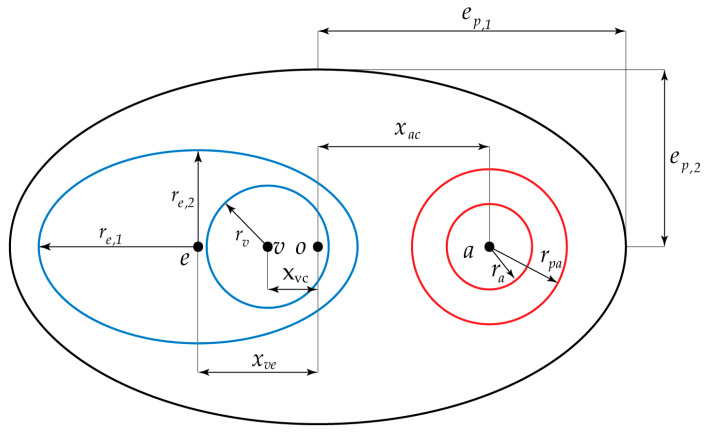
Schematic diagram of cross-sectional geometry. The marked geometric variables are the feature parameters when modelling, and all geometric lengths have been nondimensionalized using the radius of the artery $r_{a}$, and $r_{pa}$ is fixed as 2. All the dimensionless value of the parameters are listed in Table 1.

In realistic organisms, regular rounded fascia formed by loose connective tissues and regular rounded outer membranes of arteries and veins are scarce. The simplification here to a circular or elliptical shape is primarily because: (a) the effect of the relative relationship between the arteriovenous rather than the specific geometry on the flow distribution was discussed in a zero-order approximation sense; (b) the diverse flow patterns observed in the experiment were reproduced as much as possible based on the relative positions by limited shapes; (c) it avoided an introduction of subjective boundaries. The selected representative geometric parameters are shown in Figure 8 and Table 1.

In Figure 7, the circles and ellipses illustrate the cross-section of the neurovascular bundle and the topology of the PAC. Although circles cannot exactly represent the various complex patterns found in natural organisms, topological combinations of circles and ellipses have been able to functionally reproduce the experimental flow patterns, see Figure 7. For example, Figure 7A corresponds to the result that only veins are fluorescently stained, as shown in Figure 4; Figure 7C,G show that the fluorescent appears mainly in the arteriovenous space (i.e., PAC(p)) and slightly diffuses to the vein or artery, as shown in Figure 3; Figure 7D,H show that both the entire PAC(v) and PAC(p) are stained, as shown in Figure 1. It should be emphasized that the flow pattern and the various cross-section geometries do not correspond exactly to the real situation. In Figure 7A,D,H, although there are differences in the geometry and relative position relationships of the arterioles and fascia, the flow patterns presented are similar. It should be noted again, that the geometry in this simulation is only one of many possibilities, and the main purpose is to simulate the experimentally observed flow pattern, not to reproduce the exact shape of the organism species’ fascia. Importantly, the simulation results elucidate that the three flows (i.e., the flows within PAC(a), PAC(v) and PAC(p)) are sufficient to reproduce the different flow patterns observed in various experiments.

### 3.4. Heart-Orientated ISF Flow

Although the ISF flow direction is a little bit contrary to intuitions, the evidence for heart orientation is solid enough (Videos S1–S5). To strengthen the reader’s confidence, one supplementary experiment was provided (Figure S2). In the Supplementary Experiment-1, the 0.5 μL fluorescent tracer was supplied to the vascular adventitia in the middle of the exposed vessels in vivo. The image showed that the fluorescent ISF flows along the upstream venous adventitial (i.e., PAC(v) and PAC(p)), flowing toward the heart (Figure S2C,E). However, the downstream vessels of the fluorescent supplied point did not appear in the fluorescent signal (Figure S2D,F). The Supplementary Videos and Figures provide vivid and reliable evidence for the heart-orientated ISF flow.

### 3.5. The Kinematic Laws of ISF Flow along PAC around Neurovascular Bundles

In previous work [19], the three kinematic laws of ISF flow for isolated arteries and veins have been proposed. Based on the new phenomena, we took a holistic perspective of the accompanying artery, vein and nerve. The real topology structures and flow images of the PAC are highly variable in vivo. However, there is one commonality or invariance, i.e., the direction of the ISF flow remains the same in the whole process, which is supported by extensive empirical evidence. Based on such an invariance, the kinematic laws of the ISF along the PAC around neurovascular bundles were proposed.

**The First law**: There exists an ISF flow along the PAC around neurovascular bundles.

**The Second law**: Along the PAC around the neurovascular bundles, the direction of the ISF flow is the same as that of the accompanying venous blood flow and opposite to that of the accompanying arterial blood flow.

The second law shows that on the extremities, the ISF flow along the PAC is towards the heart, i.e., it is heart-orientated.

## 4. Materials and Methods

### 4.1. Experimental Animals

Thirty male BALB/c mice aged 8–10 weeks were purchased from Beijing HFK Bio-Technology Co., Ltd., (Beijing, China). The mice were anesthetized using a combination of 2.5% Avertin at 0.12–0.15 mL/10 g intraperitoneally and 0.02–0.04 mL/10 g Meloxicam subcutaneously. After the experiment, the remaining mice were executed using the overdose anesthesia method. All experiments involving animals conformed to ARRIVE (Animal Research: Reporting of In Vivo Experiments) guidelines. This study protocol was reviewed and approved by Institutional Animal Care and Use Committee, Tsinghua University (No.: 20-YYJ1).

### 4.2. Surgical Operation and Fluorescent Tracing

Surgical incisions on the mice’s legs were made from foot to groin to fully expose the vein, artery and nerve. A litter of the outer connective tissue covering the vascular adventitia was removed by forceps. 1.0–5.0 μL fluorescent sodium (Guangzhou Baiyunshan Pharmaceutical Co., Ltd., Guangzhou, China) was supplied slowly to the vascular adventitia near the foot and ankle (Figure 6).

### 4.3. Fluorescing Imaging

Observe the ISF flow through Fluorescence Stereo Microscopes ZEISS Axio Zoom V16 (ZEISS, Jena, Germany). The dynamic videos were recorded with a time series of 4s/frame for continuous filming. 

### 4.4. Histological Staining

The tissue specimens were obtained immediately after fluorescing imaging. The fixed tissues were embedded with OCT compound then 5 um slices were prepared, cutting by −20 °C freezing microtome. Immediate fluorescence images were acquired by a fluorescence microscope (Nikon, Tokyo, Japan) equipped with a charge-coupled device (CCD) (Nikon, Tokyo, Japan). The remaining sections were fixed and dehydrated according to the standard treatment of dewaxing, hydration and staining with Elastic Van Gieson (EVG). The images were acquired by an upright light microscope (Olympus, Tokyo, Japan).

### 4.5. Sample Size Calculation

Taking into account the unique design to confirm that the ISF flow along the PAC is universal in mice, traditional sample size calculations are not applicable. One thing that needs to be clarified is that such an experiment has been repeated hundreds of times in mice, and the phenomena of the ISF flow along the PAC are proved universal. In this article, to measure the diameter of PAC(a), PAC(v), and PAC(p), we used thirty 8–10 week old BALB/c mice in order to reduce errors.

## 5. Conclusions

In this study, the presence of the ISF flow was verified around the neurovascular bundles, and its flow along vascular walls was confirmed to be massive. Based on the new phenomena, the internal subspaces of PAC, i.e., PAC(a), PAC(v) and PAC(p), were proposed. Using numerical simulations, it was confirmed that different combinations of the three subspaces were sufficient to reproduce the various flow patterns observed in experiments, which indirectly validated the applicability of the PAC together with its subspaces. The kinematic laws of the ISF flow along the PAC around neurovascular bundles are proposed. We hope that the novel phenomena above may draw the attention of researchers and may be examined or checked by other laboratories.

Up until now, the physiological functions of the rapid ISF flows are still unclear. Even so, it is still reasonable to believe that such a long-range, continuous and heart-orientated ISF flow is indispensable and vital to life systems. Of course, if the rapid ISF flows are circulated, then the physiological functions may be imaginable. The findings of this study lay the foundation and open the way for establishing and developing the kinematics and dynamics of the ISF flow along the PAC around neurovascular bundles. 

## 6. Limitations

This study has not provided an accurate velocities range of the ISF flow, and has only given centimeters per second (cm/s) as the velocity magnitude. The ISF flow is high-speed compared to the lymphatic fluid flow rate (1.8–6 cm/min) [28,29]. We have tried various experimental methods to quantify the ISF flow. For example, tracking the fluorescent signal front-end through continuous images recorded by Fluorescence Stereo Microscopes has been performed. Nevertheless, the interference of uneven and variational background signal intensity to the actual fluorescence signal front-end cannot be avoided. It is also limited by the recording resolution and sensitivity to the fluorescent signal of the CCD. In addition, fluorescent microspheres used in Mestre et al. [9] and Bedussi et al. [30] were applied to track the ISF flow along the PAC without being able to form a continuous flow. We speculate that the possible reason for this is that there is a different environment for the ISF flow in the legs compared to the watery brain. Measuring the accurate velocity range of the ISF flow along the PAC is an issue for future research to explore.

## Figures and Tables

**Table 1:** Simulation parameters.

No.	Vein					Artery	PVC	
	$r_{v}$	$x_{vc}$	$e_{v,1}$	$e_{v,2}$	$x_{ve}$	$x_{ac}\;$	$e_{p,1}\;$	$e_{p,2}$
(b)	2.4	1.2	4.4	2.6	2.0	4.6	6.8	3.2
(c)	2.4	2.0	2.6	4.4	2.0	2.0	6.2	5.0
(d)	2.4	2.4	4.0	4.0	2.4	4.8	7.0	4.6
(e)	2.0	0.4	4.0	2.4	1.6	5.0	6.4	3.2
(f)	2.4	2.0	4.4	2.6	2.0	4.6	6.8	3.2
(g)	2.0	2.0	2.6	4.4	2.0	4.8	5.6	5.0
(h)	2.4	2.0	4.0	4.0	2.0	4.0	6.2	4.8

The data in the table have been nondimensionalized with respect to the arterial radius.

## Data Availability

Not applicable.

## Supplementary Materials

The following are available online at https://www.mdpi.com/article/10.3390/jfb13040172/s1, Figure S1: Diffusion of fluorescent tracers in the dead state, Figure S2: The fluorescent ISF flows towards the heart in vivo, Video S1: ISF flow along PAC(p), Video S2: Equal-width double-belt ISF flow along PAC(v), Video S3: Unequal-width double-belt ISF flow along PAC(v), Video S4: Torn fascia across the vein induced waterfall-like ISF flow, Video S5: The small vessel across the vein induced a waterfall-like ISF flow.
