# Supplementary material for: Interstitial Fluid Flows along Perivascular and Adventitial Clearances around Neurovascular Bundles
Source: J Funct Biomater. 2022 Oct 1;13(4):172. doi: 10.3390/jfb13040172 (PMC9624367; doi:10.3390/jfb13040172)
Supplement: Supplementary file 1 [file jfb-13-00172-s001.zip › Supplementary Experiments.pdf]

Supplementary Experiment-1

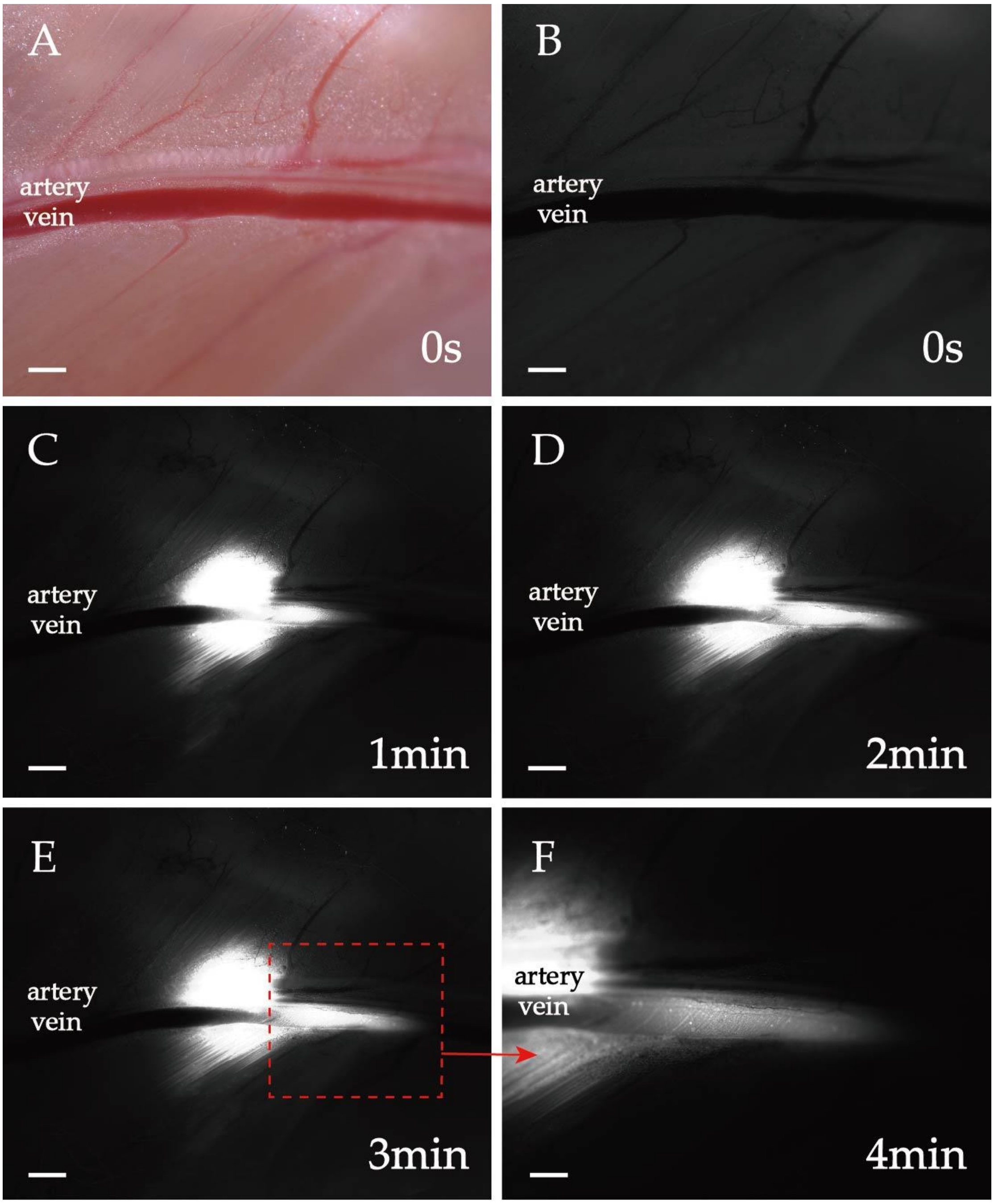

Figure S1. Diffusion of fluorescent tracers in the dead state. (**A**) Brightfield observation before fluorescing in the dead state. (**B**) Fluorescence observation before fluorescing in the dead state. (**C,D,E,F**) Fluorescence observation after fluorescing in the dead state. The heart is located on the left and the ankle is right. The fluorescent tracer diffused towards the low-lying ankle direction. Scale bar: 500 μm.

Mice were executed after being injected with an overdose of anesthesia. The femoral vein, artery, and nerve were fully exposed in the dead state (Figure S1A, S1B). The heart is located in the left direction, and the ankle is right. When the mice were immobilized supine, the ankle's height was lower than the thigh (same as all experiments shown in the manuscript). 0.5 μL fluorescent tracer was supplied slowly to the vascular adventitia in

the middle of the exposed vessels in the dead state (Figure S1C, S1D, S1E, S1F). 1min later, the fluorescent ISF has more diffused toward the low-lying ankle direction (Figure S1C). 2min and 3min later, the fluorescent front-end continued to diffuse to the ankle direction, which is far from the heart (Figure S1D, S1E). We can also clearly observe that the fluorescent tracer stained the outer connective tissue of the vein and its surrounding connective tissue in the dead state, which was quite different from the ISF flow staining in vivo (Figure S1F).

Supplementary Experiment -2

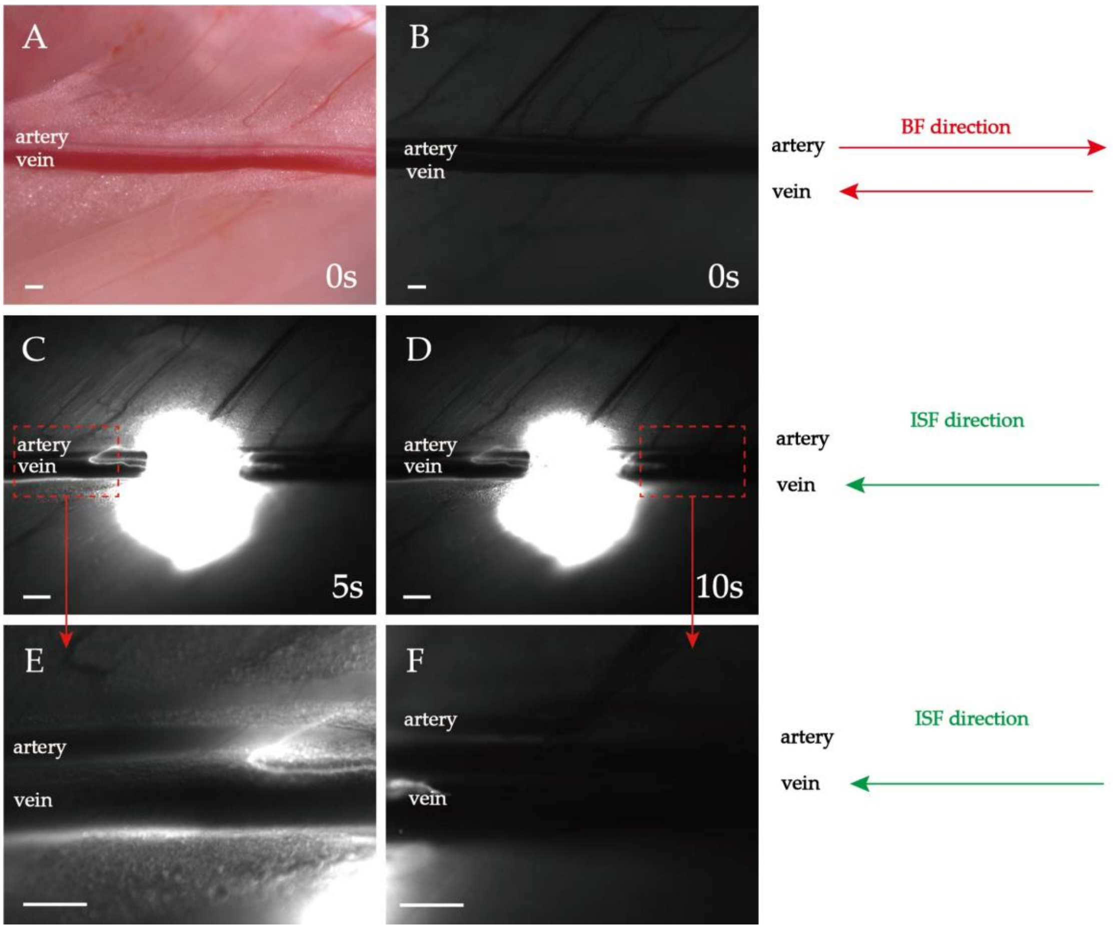

Figure S2. The fluorescent ISF flows towards the heart in vivo. (**A**) Brightfield observation before fluorescing in vivo. (**B**) Fluorescence observation before fluorescing in vivo. (**C,D,E,F**) Fluorescence observation after fluorescing in vivo. The heart is on the left. The fluorescent ISF flow is pointed to the heart. Scale bar: 500 μm.

The femoral vein, artery, and nerve were fully exposed (Figure S2A, S2B). The heart is on the left side, and the ankle is on the right. 0.5 μL fluorescent sodium was supplied slowly to the vascular adventitia in the middle of the exposed vessels in vivo (Figure S2C, S2D). 5 seconds later, the fluorescent ISF flowed towards the heart on the left side. Hence, the "linear-shaped" fluorescent pathway appeared over the venous adventitial (Figure S2C, S2E). However, the vein and artery and their surrounding connective tissue downstream of the supplying point showed no fluorescent signal (Figure S1C). 10s later,

the bright signal of the “linear-shaped” fluorescent pathway on the left side appeared to weaken. At the same time, on the right side, the fluorescent signal still did not appear (Figure S2F).

Supplementary Videos

Video S1: ISF flow along PAC(p).
Video S2: Equal-width double-belt ISF flow along PAC(v).
Video S3: Unequal-width double-belt ISF flow along PAC(v).
Video S4: Torn fascia across the vein induced waterfall-like ISF flow.
Video S5: The small vessel across the vein induced a waterfall-like ISF flow.
